# Burden of carbon monoxide poisoning in China, 1990–2019: A systematic analysis of data from the global burden of disease study 2019

**DOI:** 10.3389/fpubh.2022.930784

**Published:** 2022-07-28

**Authors:** Peng Cui, Yan Jin, Huaizhi Feng, Zhitao Li, Shuangning Ding, Yongze Li

**Affiliations:** ^1^Department of Interventional Radiology, Chengdu Municipal Third People's Hospital, Chengdu, China; ^2^Department of Emergency, The First Hospital of China Medical University, Shenyang, China; ^3^Department of Emergency, Shenyang Orthopedic Hospital, Shenyang, China; ^4^Department of Psychiatry and Psychological Clinic, Affiliated Quanzhou First Hospital, Fujian Medical University, Quanzhou, China; ^5^National Health Commission (NHC), Key Laboratory of Diagnosis and Treatment of Thyroid Diseases, Department of Endocrinology and Metabolism, Institute of Endocrinology, The First Hospital of China Medical University, Shenyang, China

**Keywords:** carbon monoxide poisoning, epidemiology, China, disability-adjusted life years (DALYs), mortality, public health

## Abstract

**Background:**

Carbon monoxide (CO) poisoning is one of the most common toxic occupational diseases, but related data in China are scarce. A better understanding of the burden of CO poisoning is essential for improving its management.

**Methods:**

A systematic analysis of data from the Global Burden of Disease (GBD) Study 2019 was conducted. Following the general analytical strategy used in the GBD Study 2019, the sex- and age-specific incidence and mortality rates of CO poisoning and disability-adjusted life years (DALYs) due to CO poisoning in China were analyzed. Estimated average annual percentage changes (AAPCs) in age-standardized rates were calculated by joinpoint regression analysis. The effects of age, period and cohort on the incidence of CO poisoning and DALYs due to CO poisoning were estimated by an age-period-cohort model.

**Results:**

The age-standardized incidence and mortality rates as well as DALYs of CO poisoning per 100,000 population were estimated to be 21.82 [95% uncertainty interval (UI): 15.05–29.98], 0.93 (95% UI: 0.63–1.11), and 40.92 (95% UI: 28.43–47.85), respectively, in 2019. From 1990 to 2019, the AAPCs in the age-standardized incidence significantly increased in both males and females, while the age-standardized mortality rates and DALYs significantly decreased in both males and females. The incidence of CO poisoning peaked in individuals aged 15–19 years. Males had a higher burden of CO poisoning than females. The age effect showed that the relative risks (RRs) of incident CO poisoning decreased with age among males and females and that individuals aged 15–24 years had the highest RRs. The RRs of incident CO poisoning increased with time. The cohort effect showed that the incidence increased in successive birth cohorts.

**Conclusions:**

The incidence of CO poisoning in China increased from 1990 to 2019. More attention should be given to improving the burden of CO poisoning in Chinese adolescents. The results of this study can be used by health authorities to inform preventative measures to reduce the burden of CO poisoning.

## Background

Carbon monoxide (CO) poisoning is one of the most common causes of fatal poisoning worldwide. Long-term exposure to potentially lethal doses of CO exceeding 100 ppm can cause poisoning; CO is produced by internal combustion engines, fossil fuel furnaces and fire and is toxic (1). Although the CO emissions of modern cars are controlled by regulatory standards, they remain highly toxic in poorly ventilated environments. CO poisoning is the most common type of accidental poisoning. Approximately 970,000 poisoning incidents occur every year worldwide, and ~41,000 people die each year from CO poisoning (2).

In many economically developed Western countries, electricity is the main clean energy source; thus, the risk of CO poisoning is reduced. However, in less-developed countries, such as China, CO poisoning is still a widespread public health problem (3). China's economy has developed rapidly in the past two decades. Unfortunately, this vigorous development has not been very balanced. Therefore, the characteristics of CO poisoning in China differ from those in Western countries. In Western countries, the main causes of CO poisoning are weather conditions unfavorable to CO removal, insufficient fireplace ventilation, insufficient combustion ventilation, and increased vehicle exhaust within limited spaces (4). However, in China, the primary cause of CO poisoning is indoor heating and occupational exposure (5, 6).

A greater understanding of risk profiles and onset patterns associated with CO poisoning could facilitate the early identification of individuals at risk of CO poisoning, thereby supporting timely interventions that could effectively reduce the CO poisoning burden. However, studies concerning the CO poisoning burden in China are scarce (6–10). These studies are limited to certain regions and specific populations, and detailed analyses of temporal trends and age- and sex-specific differences have not been performed.

Recently, the Global Burden of Disease (GBD) Study group published updated GBD estimations, providing a standardized methodology to estimate the disease burden of CO poisoning by age, sex, year, and location (11). As the first step in evaluating CO poisoning in China, we used data from the GBD Study 2019 to describe the age-standardized incidence rate (ASIR), age-standardized mortality rate (ASMR), and disability-adjusted life years (DALYs) of CO poisoning in 2019. We also examined the temporal trends of CO poisoning in China from 1990 to 2019 by using joinpoint regression analysis. In addition, we further explored the net age, period, and cohort effects under the age-period-cohort framework.

## Methods

### Data source

The GBD Study 2019, which covered 204 countries and regions from 1990 to 2019, provides a comprehensive assessment of health burden for 369 diseases and injuries (11). Details of the methodology used in the GBD Study 2019 are described on the official website (http://ghdx.healthdata.org/gbd-results-tool). The GBD Study 2019 used systematic reviews, survey data, hospital data, disease registries, inpatient and outpatient data, claims, and case notifications as data sources to estimate disease incidence. The raw epidemiological data that were used to calculate the prevalence estimates met strict inclusion criteria, such as being representative of the general population and therefore not including any treatment or clinical samples. Population estimates and confidence intervals were produced by Bayesian statistical methods. In these data, DALYs are the sum of years lived with disability (YLDs) and years of life lost (YLLs). YLLs represent the product of the number of deaths and the remaining life expectancy considering the standard optimal age at death. Standard life expectancy is derived from the lowest observed death rate by age in any population of more than 5 million people worldwide. YLDs represent the prevalence of individual consequences of the disease (or sequelae) multiplied by the product of its corresponding disability weight, which quantifies the relative severity of the sequelae as a number between 0 (representing complete health) and 1 (representing death). The datasets used in the present study can be found on the GBD website at http://ghdx.healthdata.org/gbd-results-tool. We abstracted the data and further conducted a systematic analysis of this topic.

### Case definition

According to the disease and injury classifications of the GBD Study 2019, CO poisoning was defined as codes X47-X47.9 and J70.5 in the International Classification of Diseases and Injuries, 10th edition (ICD-10) (11). Under this definition, “CO poisoning” includes accidental poisoning from and exposure to CO from internal combustion engine exhaust, utility gas, other domestic fuels, other sources and unspecified sources, as well as accidental poisoning by and exposure to other specified gases or vapors and unspecified gases or vapors. Importantly, exposure to CO due to self-poisoning or undetermined intent (ICD-10 code: Z91.5) was excluded.

### Statistical analysis

Joinpoint regression analysis was used to assess trends in the disease burden of CO poisoning. JoinPoint software (Joint Command Line, version 4.5.0.1) was provided by the Surveillance Research Program of the National Cancer Institute (12). Joinpoint regression analysis analyses temporal trends in data and then fits the simplest model possible to the data by connecting several different line segments on a logarithmic scale. Annual percentage change (APC) is one way to characterize trends in disease rates over time. The APC was calculated using the geometrically weighted average of the various APC values in the regression analysis (12). The average annual percentage change (AAPC) is a summary of the trend over a pre-specified fixed interval. The AAPC provides a single number that describes the average APCs over a period of multiple years and is valid even if the joinpoint model indicates changes in trends during those years (12). This value is computed as a weighted average of the APCs from the joinpoint model, with the weights equal to the length of the APC interval. The age-standardized rates and their AAPCs were calculated to assess the incidence and mortality rates of CO poisoning and DALYs due to CO poisoning using linear regression analysis. APCs were calculated to assess trends, and the *Z*-test was used to assess whether the APC was significantly different from zero (12). When describing trends, the terms increase or decrease are used when the slope of the trend is significantly different from zero (12).

To assess risk in a population in a given year and the accumulation of health risks since birth, an age-period-cohort model was constructed (13). The age-period-cohort model provides a useful parametric framework that complements the standard non-parametric descriptive approach. The model allowed analysis of the independent effects of age, period, and cohort on temporal trends in CO poisoning. In this model, the data collected were divided into consecutive 5-year age groups and consecutive 5-year periods. The incidence of CO poisoning was recorded in consecutive 5-year age groups (0–4 to 75–79 years), in consecutive 5-year periods (1994–2019), and in corresponding consecutive 5-year birth cohorts from 1915–1919 to 2015–2019. Age-period-cohort analyses using intrinsic estimation methods provided estimated coefficients for the age, period, and cohort effects. These coefficients were transformed into exponential values that denote the relative risk of the incidence in a particular age, period, or birth cohort relative to the average level of all ages, periods, or birth cohorts combined. The age-period-cohort analysis was performed using STATA 15.0 software (StataCorp, College Station, TX, USA). All the rates are reported per 100,000 population. The 95% uncertainty interval (UI) for each variable was calculated in our study. Significance in all the analyses was assessed at the 0.05 level, and all hypothesis tests were two-sided.

## Results

### CO poisoning burden in China in 2019

The ASIR and ASMR of CO poisoning and the age-standardized DALYs due to CO poisoning in 2019 according to sex are presented in Table 1. The ASIR [23.31 (95% UI: 16.33–31.63) vs. 20.25 (95% UI: 13.85–28.33) per 100,000 population], ASMR [1.16 (95% UI: 0.59–1.49) vs. 0.72 (95% UI: 0.49–0.92) per 100,000 population], and age-standardized DALYs [49.92 (95% UI: 26.59–63.35) vs. 31.70 (95% UI: 22.26–39.47) per 100,000 population] were higher among males than among females.

**Table 1:** Trends in the burden of carbon monoxide poisoning between males and females in China from 1990 to 2019.

**Variable**	**Rate in 1990 (95% UI)**	**Rate in 2019 (95% UI)**	**AAPC, % (95% CI)**
All
Incidence	16.20 (11.08–22.45)	21.82 (15.05–29.98)	1.4 (1.1–1.6)
Mortality	1.18 (0.98–1.58)	0.93 (0.63–1.11)	−0.4 (−0.8 to −0.1)
DALYs	62.91 (50.61–82.60)	40.92 (28.43–47.85)	−1.4 (−1.7 to −1.1)
Males
Incidence	16.73 (11.68–23.04)	23.31 (16.33–31.63)	1.2 (1.1–1.3)
Mortality	1.51 (1.16–2.26)	1.16 (0.59–1.49)	−0.9 (−1.2 to −0.6)
DALYs	75.38 (54.88–108.62)	49.92 (26.59–63.35)	−1.3 (−1.6 to −1.1)
Females
Incidence	15.67 (10.52–22.07)	20.25 (13.85–28.33)	0.9 (0.8–1.0)
Mortality	0.88 (0.66–1.2)	0.72 (0.49–0.92)	−0.7 (−1.1 to −0.3)
DALYs	50.12 (35.45–69.45)	31.7 (22.26–39.47)	−1.6 (−2.2 to −0.9)

*UI, uncertainty interval; AAPC, average annual percentage change; CI, confidence interval*.

### Trends in CO poisoning burden from 1990 to 2019

**Figure 1:**
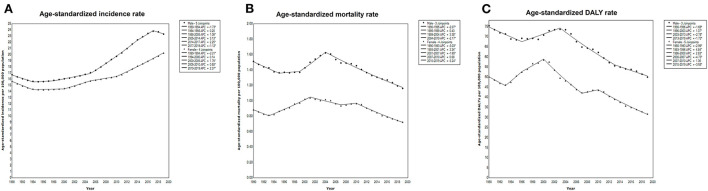
Joinpoint regression analysis of the age-standardized **(A)** incidence, **(B)** mortality, and **(C)** DALYs due to CO poisoning according to sex in China from 1990 to 2019.

As shown in Figure 1 and Table 1, the ASIR increased and the ASMR and age-standardized DALYs decreased from 1990 to 2019 in China. Among both males and females, the ASIR significantly increased with an AAPC of 1.4 (95% CI: 1.1–1.6), but the ASMR (AAPC: −0.4; 95% CI: −0.8 to −0.1) and age-standardized DALYs (AAPC: −1.4; 95% CI: −1.7 to −1.1) decreased from 1990 to 2019. The AAPC in the ASIR [1.2% (95% CI: 1.1–1.3%) vs. 0.9% (95% CI: 0.8–1.0%)] was significantly higher in males than in females. Joinpoint regression analysis further identified the most recent decade as a timepoint with a significant decrease in the ASMR and age-standardized DALYs (Figure 1).

### CO poisoning burden by age and sex in 2019

**Figure 2:**
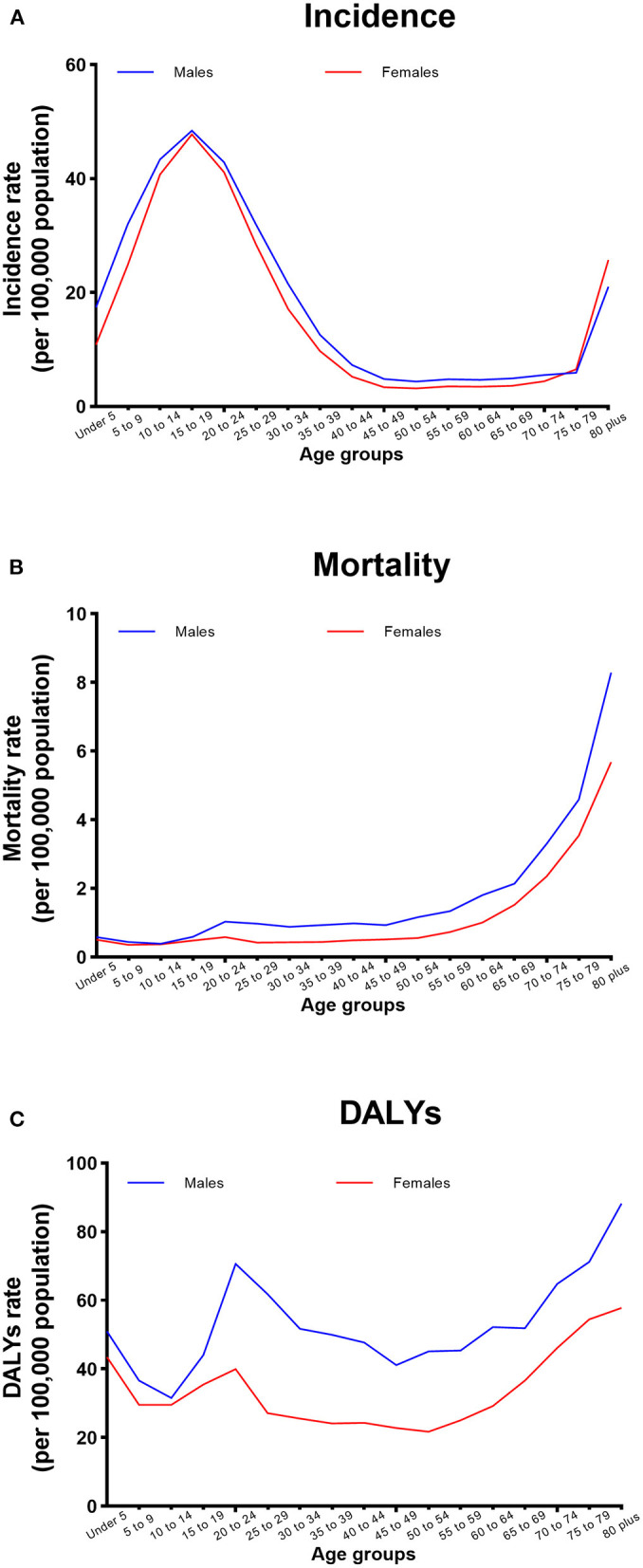
Sex-specific **(A)** incidence and **(B)** mortality rates of CO poisoning and **(C)** DALYs due to CO poisoning according to age in China in 2019.

The burden of CO poisoning for males and females was similar with increasing age, and males had a higher burden than females from under 5 years to 80 years (Figure 2).

### Age-period-cohort analysis with the intrinsic estimator method

The estimated RRs for CO poisoning and associated DALYs due to age, period, and cohort effects are shown in Table 2. After controlling for period and cohort effects, the net age effect showed that the RR of CO poisoning significantly increased among the 5–34 years group in both males and females. During the period of observation, the RR of CO poisoning increased among males and females after 2014, although there was no significant difference (Table 2). The RR for DALYs associated with period effects showed a significant increase in 2004 (RR: 1.14; 95% CI: 1.08–1.21) in males and significant increases (RR: 1.09; 95% CI: 1.02–1.17 and RR: 1.09; 95% CI: 1.02–1.17) in 1999 and 2004, respectively, for females (Table 2). Regarding the cohort effect, the RRs of CO poisoning significantly increased in later birth cohorts (1985–1989, 1990–1994, 1995–1999, 2000–2004, 2005–2009, and 2010–2014) in both males and females (Table 2).

**Table 2:** Sex-specific relative risks of carbon monoxide poisoning and DALYs due to carbon monoxide poisoning in China due to age, period, and cohort effects.

**Factor**	**Incidence in males**		**Incidence in females**		**DALYs in males**		**DALYs in females**	
	**RR (95% CI)**	***P*** **-value**	**RR (95% CI)**	***P*** **-value**	**RR (95% CI)**	***P*** **-value**	**RR (95% CI)**	***P*** **-value**
Age (years)
0–4	1.09 (0.84–1.43)	0.507	0.89 (0.64–1.24)	0.502	2.39 (2.18–2.62)	<0.001	3.7 (3.33–4.1)	<0.001
5–9	1.74 (1.42–2.14)	<0.001	1.95 (1.55–2.45)	<0.001	0.96 (0.86–1.08)	0.51	1.35 (1.2–1.53)	<0.001
10–14	2.31 (1.94–2.76)	<0.001	3.2 (2.65–3.85)	<0.001	0.69 (0.61–0.78)	<0.001	0.98 (0.87–1.12)	0.817
15–19	2.86 (2.43–3.36)	<0.001	3.96 (3.34–4.7)	<0.001	0.83 (0.75–0.93)	0.001	0.97 (0.85–1.1)	0.641
20–24	3.05 (2.59–3.59)	<0.001	3.54 (2.96–4.24)	<0.001	1.01 (0.91–1.12)	0.844	1.03 (0.91–1.16)	0.684
25–29	2.56 (2.13–3.08)	<0.001	2.53 (2.05–3.13)	<0.001	0.89 (0.8–1)	0.041	0.8 (0.69–0.92)	0.001
30–34	1.67 (1.34–2.1)	<0.001	1.6 (1.24–2.07)	<0.001	0.82 (0.73–0.93)	0.001	0.73 (0.63–0.84)	<0.001
35–39	0.98 (0.74–1.3)	0.876	0.91 (0.66–1.26)	0.564	0.85 (0.75–0.95)	0.005	0.65 (0.55–0.76)	<0.001
40–44	0.63 (0.45–0.88)	0.007	0.52 (0.34–0.78)	0.002	0.9 (0.8–1)	0.058	0.63 (0.54–0.74)	<0.001
45–49	0.42 (0.28–0.64)	<0.001	0.35 (0.21–0.57)	<0.001	0.81 (0.72–0.91)	0.001	0.65 (0.55–0.76)	<0.001
50–54	0.38 (0.25–0.59)	<0.001	0.34 (0.2–0.56)	<0.001	0.84 (0.75–0.94)	0.004	0.62 (0.53–0.73)	<0.001
55–59	0.44 (0.29–0.67)	<0.001	0.39 (0.24–0.64)	<0.001	0.87 (0.78–0.98)	0.021	0.71 (0.61–0.83)	<0.001
60–64	0.46 (0.31–0.71)	<0.001	0.42 (0.26–0.69)	0.001	0.99 (0.89–1.11)	0.901	0.81 (0.7–0.94)	0.007
65–69	0.54 (0.36–0.8)	0.003	0.5 (0.31–0.8)	0.004	1.04 (0.94–1.16)	0.439	1.13 (0.98–1.29)	0.086
70–74	0.68 (0.46–0.99)	0.046	0.66 (0.42–1.02)	0.064	1.35 (1.22–1.49)	<0.001	1.6 (1.41–1.81)	<0.001
75–79	0.82 (0.56–1.21)	0.322	1.07 (0.71–1.62)	0.754	1.61 (1.45–1.79)	<0.001	2.07 (1.81–2.36)	<0.001
Period
1994	0.95 (0.82–1.1)	0.47	0.97 (0.82–1.14)	0.707	1.02 (0.96–1.08)	0.484	0.95 (0.88–1.03)	0.224
1999	0.93 (0.81–1.06)	0.265	0.93 (0.8–1.08)	0.321	0.98 (0.93–1.04)	0.57	1.09 (1.02–1.17)	0.017
2004	0.93 (0.82–1.05)	0.254	0.96 (0.84–1.09)	0.534	1.14 (1.08–1.21)	<0.001	1.09 (1.02–1.17)	0.012
2009	1.01 (0.9–1.13)	0.909	0.97 (0.85–1.1)	0.657	1.05 (0.99–1.11)	0.116	1.06 (0.98–1.13)	0.145
2014	1.11 (0.98–1.25)	0.096	1.05 (0.91–1.2)	0.499	0.94 (0.89–1)	0.059	0.96 (0.89–1.04)	0.293
2019	1.1 (0.95–1.27)	0.186	1.14 (0.97–1.34)	0.109	0.88 (0.83–0.94)	<0.001	0.87 (0.8–0.94)	0.001
Cohort
1915–1919	0.56 (0.23–1.36)	0.201	0.38 (0.13–1.1)	0.074	0.79 (0.63–0.99)	0.044	0.54 (0.39–0.74)	<0.001
1920–1924	0.61 (0.32–1.16)	0.133	0.47 (0.22–1.03)	0.06	0.84 (0.71–1)	0.048	0.58 (0.46–0.73)	<0.001
1925–1929	0.65 (0.37–1.12)	0.121	0.58 (0.31–1.1)	0.094	0.91 (0.79–1.05)	0.19	0.78 (0.66–0.93)	0.007
1930–1934	0.67 (0.41–1.09)	0.107	0.67 (0.39–1.17)	0.164	0.98 (0.87–1.1)	0.708	0.92 (0.79–1.07)	0.277
1935–1939	0.68 (0.43–1.06)	0.086	0.74 (0.45–1.22)	0.243	1 (0.89–1.12)	0.973	0.99 (0.86–1.14)	0.892
1940–1944	0.71 (0.47–1.08)	0.11	0.78 (0.5–1.24)	0.295	1 (0.9–1.12)	0.964	1.02 (0.89–1.17)	0.816
1945–1949	0.8 (0.52–1.23)	0.319	0.88 (0.53–1.45)	0.615	1.08 (0.97–1.21)	0.169	1.17 (1.01–1.36)	0.038
1950–1954	0.89 (0.58–1.37)	0.607	0.95 (0.57–1.58)	0.842	1.14 (1.02–1.29)	0.027	1.29 (1.1–1.51)	0.002
1955–1959	0.98 (0.66–1.47)	0.931	1.04 (0.64–1.67)	0.886	1.19 (1.05–1.34)	0.006	1.27 (1.07–1.51)	0.006
1960–1964	1.04 (0.72–1.49)	0.838	1.11 (0.73–1.7)	0.621	1.18 (1.04–1.34)	0.009	1.33 (1.12–1.58)	0.001
1965–1969	1.07 (0.78–1.47)	0.666	1.16 (0.81–1.67)	0.425	1.19 (1.05–1.35)	0.006	1.42 (1.2–1.68)	<0.001
1970–1974	1.07 (0.82–1.4)	0.615	1.19 (0.87–1.61)	0.275	1.18 (1.04–1.33)	0.01	1.39 (1.19–1.63)	<0.001
1975–1979	1.1 (0.87–1.39)	0.42	1.21 (0.94–1.56)	0.14	1.22 (1.09–1.38)	0.001	1.31 (1.12–1.53)	0.001
1980–1984	1.19 (0.98–1.45)	0.084	1.29 (1.04–1.6)	0.019	1.29 (1.15–1.45)	<0.001	1.33 (1.16–1.54)	<0.001
1985–1989	1.26 (1.06–1.5)	0.008	1.32 (1.1–1.59)	0.003	1.31 (1.17–1.46)	<0.001	1.42 (1.25–1.61)	<0.001
1990–1994	1.35 (1.15–1.57)	<0.001	1.39 (1.18–1.65)	<0.001	1.3 (1.18–1.43)	<0.001	1.47 (1.32–1.63)	<0.001
1995–1999	1.43 (1.21–1.69)	<0.001	1.43 (1.2–1.71)	<0.001	1.32 (1.2–1.46)	<0.001	1.4 (1.26–1.56)	<0.001
2000–2004	1.51 (1.25–1.84)	<0.001	1.44 (1.16–1.77)	0.001	1.01 (0.89–1.13)	0.92	0.98 (0.86–1.12)	0.779
2005–2009	1.55 (1.22–1.97)	<0.001	1.46 (1.12–1.91)	0.006	0.74 (0.63–0.86)	<0.001	0.72 (0.6–0.85)	<0.001
2010–2014	1.52 (1.1–2.09)	0.011	1.45 (1–2.12)	0.04	0.59 (0.49–0.71)	<0.001	0.58 (0.47–0.71)	<0.001
2015–2019	1.48 (0.86–2.55)	0.152	1.46 (0.74–2.87)	0.27	0.45 (0.34–0.6)	<0.001	0.42 (0.31–0.58)	<0.001

*RR denotes the relative risk of incidence or DALYs in a particular age, period, or birth cohort relative to the average level in all ages, periods, or birth cohorts. RR, relative risk; CI, confidence interval*.

## Discussion

To our knowledge, the present study is the first comprehensive evaluation of the considerable and continuously increasing trends in the incidence of CO poisoning in China from 1990 to 2019 using joinpoint regression and an age-period-cohort framework based on data from the GBD Study 2019. The standardized methods for estimating CO poisoning metrics in the GBD Study 2019 made it possible to compare regional and global metrics with those at the national level in China. In addition, the present study is also the first report of DALYs due to CO poisoning in China. These comprehensive and reliable data are important for policymakers, as they can inform policies to prevent CO poisoning and improve the quality of life of patients.

In 2019, there were 0.27 million prevalent cases and 0.24 million incident cases of CO poisoning, 0.02 million deaths, and 0.58 million DALYs due to CO poisoning (0.02 million YLDs and 0.56 million YLLs) in China (14). The burden of CO poisoning in China is higher than that in middle sociodemographic index (SDI) regions and that worldwide. This difference can be partly explained by differences in economic structures and sociohistorical factors in China (3). In underdeveloped areas, especially remote rural areas in northern China, local residents prefer to use honeycomb briquettes for household heating. Therefore, accidents related to events such as chimney blockages or poor ventilation occur frequently, resulting in high rates of CO poisoning. Previous studies have examined the correlation between CO poisoning parameters and the SDI and found that a lower SDI score is associated with a higher mortality rate (2). Therefore, cost is important to consider in the research and development of new CO treatment technologies. An explanation for this phenomenon may be related to the political economy, as research suggests that the economy and policies associated with SDI scores are related to the incidence of certain diseases (15). However, given the very limited information on this issue, further studies in China are needed to explore potential explanations.

The CO poisoning burden varied by age and sex in the present study. Regarding sex, we found that the burden of CO poisoning was higher in males than in females, which is consistent with previous studies (2, 9). These differences persisted across age groups and calendar years. An important explanation for this phenomenon may be the difference in exposure between the sexes. Previous studies have suggested that occupations with a higher risk of CO poisoning, including jobs that require proximity to combustion sources (such as engines and fire) or CO-emitting equipment (such as firefighting equipment, diesel and forklift engines, and mechanical equipment), have a higher rate of male employees (3). Because of the nature of these jobs, they are more suitable for males. Thus, males may be exposed to more risk factors for CO poisoning than females. Similar to previous studies that analyzed CO poisoning at the global level, the peak age for incident CO poisoning was 15–19 years (2). Generally, the age effect explained why the incidence decreased with increasing age among males and females. These results indicate that in China, infants (aged 0–4 years) and older individuals (aged 70 years and older) had a higher risk of DALYs, while younger individuals (aged 15–24 years) had a higher risk of CO poisoning. Many victims or potential victims in China lack basic first aid knowledge regarding CO poisoning, such as accelerating respiration and moving away from areas with high concentrations of CO, making them more susceptible to this disease (3). Thus, education about prevention is required to decrease the rate of CO poisoning among adolescents.

Period effects are usually due to a series of complex historical events and environmental factors. We observed a significantly decreased risk of CO poisoning among males and females in 2019. This decrease may be due to the popularity of hyperbaric oxygen therapy and the development of new technologies (16). However, general understanding of the preferred treatment for CO, hyperbaric oxygen therapy, is lacking. Not only the general public but also some nursing and medical staff lack a basic understanding of this treatment method, which may affect the prognosis of patients with CO poisoning (3). Therefore, education regarding hyperbaric oxygen therapy as a treatment strategy should be implemented.

The cohort effect on the incidence of CO poisoning revealed continuously increasing trends in later birth cohorts in both males and females. This increase may be due to the increase in accidental CO poisoning in homes as heaters have become more accessible due to improvements in economic conditions. Furthermore, with the transformation of infrastructure, supplements with highly concentrated CO gas, as opposed to burning coal or charcoal, may impose a higher risk of CO poisoning due to potential gas leakage (9). Therefore, the implementation of safety standards for home heating systems and government oversight may be useful management strategies to reduce the incidence of CO poisoning (17).

Our study enhances awareness of this important public health issue; it identified risk factors for CO poisoning that can be used to inform public health actions. However, our study has several limitations. First, exposure to CO due to self-poisoning or undetermined intent was not included; this contrasts with most studies from other countries where suicidal poisoning (by car exhaust or charcoal) is one of the leading causes of diagnosed CO poisoning. Second, our study has an ecological fallacy and unique limitations associated with the age-period-cohort model (including the identifiability problem and uncertainty principle). Third, as the data for this study were derived from the GBD Study 2019, all the general limitations ascribed to the study's methodologies also apply here (11). For example, underreporting and the misdiagnosis of CO poisoning may have affected the quality of the GBD data.

## Conclusion

In conclusion, the age-standardized incidence rate of CO poisoning increased, but the associated DALYs decreased in China among both males and females during the 1990–2019 period. Notably, males had a higher burden of CO poisoning than females. In addition, younger age was associated with the highest risk of incident CO poisoning. An educational campaign addressing the many causes and circumstances of CO poisoning will benefit prevention, especially in adolescents.

## Data Availability Statement

Publicly available datasets were analyzed in this study. This data can be found at: http://ghdx.healthdata.org/gbd-results-tool.

## Author contributions

YL and PC contributed to the data acquisition, analysis, interpretation, drafted, and critically reviewed the manuscript for intellectual content. SD and ZL contributed to the data analysis. YJ and HF conceived and designed the study. YL is the guarantor of this work and, as such, had full access to all the data in the study and takes responsibility for the integrity of the data and the accuracy of the data analysis. All authors reviewed and approved the final version of the manuscript. All authors contributed to the article and approved the submitted version.

## Funding

This work was supported by the National Natural Science Foundation of China (Grant No. 82000753) and the China Postdoctoral Science Foundation (Grant No. 2021MD703910). The funding sources had no role in the study design, data analysis, interpretation, or decision to submit for publication.

## Conflict of interest

The authors declare that the research was conducted in the absence of any commercial or financial relationships that could be construed as a potential conflict of interest.

## Publisher's note

All claims expressed in this article are solely those of the authors and do not necessarily represent those of their affiliated organizations, or those of the publisher, the editors and the reviewers. Any product that may be evaluated in this article, or claim that may be made by its manufacturer, is not guaranteed or endorsed by the publisher.

## Abbreviations

CO: carbon monoxide
GBD: global burden of disease
DALYs: disability-adjusted life years
AAPCs: average annual percentage changes
ASIR: age-standardized incidence rate
ASMR: age-standardized mortality rate
YLDs: years lived with disability
YLLs: years of life lost
UI: uncertainty interval
ICD: International Classification of Diseases and Injuries.
